# Ultrathin Silicon Nanowires for Optical and Electrical Nitrogen Dioxide Detection

**DOI:** 10.3390/nano11071767

**Published:** 2021-07-07

**Authors:** Dario Morganti, Antonio Alessio Leonardi, Maria José Lo Faro, Gianluca Leonardi, Gabriele Salvato, Barbara Fazio, Paolo Musumeci, Patrizia Livreri, Sabrina Conoci, Giovanni Neri, Alessia Irrera

**Affiliations:** 1 CNR-IPCF, Istituto per i Processi Chimico-Fisici, Viale F. Stagno D’Alcontres 37, 98158 Messina, Italy; dario.morganti@ct.infn.it (D.M.); antonio.leonardi@dfa.unict.it (A.A.L.); gabriele.salvato@cnr.it (G.S.); barbara.fazio@cnr.it (B.F.); 2 Dipartimento di Fisica e Astronomia, Università di Catania, Via Santa Sofia 64, 95123 Catania, Italy; mariajose.lofaro@dfa.unict.it (M.J.L.F.); Paolo.musumeci@ct.infn.it (P.M.); 3 CNR-IMM UoS Catania, Istituto per la Microelettronica e Microsistemi, Via Santa Sofia 64, 95123 Catania, Italy; 4 Institute of Advanced Technologies for Energy (ITAE)—CNR, Salita Santa Lucia Sopra Contesse 5, 98126 Messina, Italy; leonardis@unime.it; 5 Department of Engineering, University of Palermo, Viale delle Scienze Ed.9, 90128 Palermo, Italy; patrizia.livreri@gmail.com; 6 Dipartimento di Scienze Chimiche, Biologiche, Farmaceutiche, ed Ambientali, Università Degli Studi di Messina, Viale Ferdinando Stagno d’Alcontres, 98166 Messina, Italy; sabrina.conoci-ext@st.com; 7 Dipartimento di Ingegneria, Università Degli Studi di Messina, C.da Di Dio, 98166 Messina, Italy; gneri@unime.it

**Keywords:** silicon nanowires, gas sensing, light-emission, nitrogen dioxide

## Abstract

The ever-stronger attention paid to enhancing safety in the workplace has led to novel sensor development and improvement. Despite the technological progress, nanostructured sensors are not being commercially transferred due to expensive and non-microelectronic compatible materials and processing approaches. In this paper, the realization of a cost-effective sensor based on ultrathin silicon nanowires (Si NWs) for the detection of nitrogen dioxide (NO_2_) is reported. A modification of the metal-assisted chemical etching method allows light-emitting silicon nanowires to be obtained through a fast, low-cost, and industrially compatible approach. NO_2_ is a well-known dangerous gas that, even with a small concentration of 3 ppm, represents a serious hazard for human health. We exploit the particular optical and electrical properties of these Si NWs to reveal low NO_2_ concentrations through their photoluminescence (PL) and resistance variations reaching 2 ppm of NO_2_. Indeed, these Si NWs offer a fast response and reversibility with both electrical and optical transductions. Despite the macro contacts affecting the electrical transduction, the sensing performances are of high interest for further developments. These promising performances coupled with the scalable Si NW synthesis could unfold opportunities for smaller sized and better performing sensors reaching the market for environmental monitoring.

## 1. Introduction

The interest of the scientific community and industry in gas detection has continuously become more important since the industrial revolution. In fact, the birth of the industry was followed by an exponential production of different toxic gas such as CO, CO_2_, NO_x_, NH_3_, and several hydrocarbon compounds. As a consequence, due to the effects of these toxic gases on human health, gas detection started to become a priority demand. Nowadays, gas sensors play an important role in many applications, from industrial air quality monitoring to novel automotive, and smart city applications [1,2,3,4,5,6]. Indeed, air quality control is crucial for several industrial fields such as refineries, pharmaceutical manufacturing, fumigation facilities, paper pulp mills, aircraft, hazmat operations, agriculture, and both shipbuilding and waste-water treatment facilities [7,8,9]. Among all the toxic compounds, a special role is occupied by CO and NO_2_. The risk related to CO is commonly well known, indeed it is a hemotoxin molecule that poisons the red globule cells as it strongly binds to the iron ion (Fe^2+^) in the hemoglobin instead of oxygen, causing a serious risk for health even in a small quantity of a few tens of ppm. Between all seven nitrogen oxides in the air, nitric oxide (NO) and nitrogen dioxide (NO_2_) are the main two associated with combustion sources’ process in industrial factories as well as in automotive engines, especially in diesel ones. The environmental concentrations of these two gases are variable but can exceed a total concentration of 500 μg/m^3^ (0.3 ppm) in dense urban areas [10]. Although 90–95% of nitrogen oxides are usually emitted as NO, it is rapidly oxidized in the air to NO_2_ from the available environmental oxidants. The rapid oxidation rate is such that nitrogen dioxide is generally considered the most important pollutant. It must be pointed out that the major producer of NO_2_ is nature itself. It is mainly produced through bacterial, volcanic, and lightning actions. However, these emissions are distributed over the entire surface of the earth. Therefore, the main source of risk for this gas derives from uncontrolled anthropogenic emissions through the combustion of fossil fuels [11,12,13,14].

The dangerous effects of NO_2_ on human health are known and vary based on the concentration and length of time you are exposed to. This gas can lead to deterioration and olfactive paralysis [15], even with daily exposure of about 3 ppm prolonged for more than 8 h. In confined environments such as automotive cabins, garage parking, or tunnels, high concentrations (>3 ppm) are a serious health hazard. However, the vast majority of lung biochemical studies show effects only after acute or subchronic exposure to levels of NO_2_ exceeding 3160 μg/m^3^ (~2 ppm) [11], which becomes a crucial value for the sensing of this gas.

In this scenario, the realization of cost-effective, selective, sensitive, and reliable gas sensing platforms for the detection of NO_2_ (and in general NO_x_) is an open challenge. The development of recent miniaturization techniques has contributed to the applications of nanostructures. The particular properties of nanostructured materials are increasingly attracting interest from the whole scientific community [16,17,18,19,20,21,22,23,24]. This strong interest has already brought to light the production of numerous sensing devices used in the most varied application fields, such as biological, chemical, environmental, and biomedical [25,26,27,28,29,30,31,32]. The use of nanostructures for the realization of sensors is a topic particularly investigated by the scientific community in recent years. Indeed, their very high surface-to-volume ratio (S/V) considerably enhances their interaction with the surrounding environment and, therefore, their sensing performances. Over the years, sensors based on different types of nanomaterials have been developed as novel resources for several applications surpassing the standard sensor limit of detection (LOD) [33,34,35]. Nonetheless, the common high cost and the fact that the fabrication processes and materials are not compatible with the microelectronics industry have limited the pervasion and commercial transfer of these nanostructured sensing solutions.

The design of a silicon nanostructured-based sensor may couple the nanostructure’s high performances with a large-scale production availability with a tremendous effect on the sensor market. Indeed, silicon is the leading material in the industrial sector, thanks to its characteristics of availability (it is a very abundant element on the earth’s surface), low-cost, integrability, and ease of preparation. This makes the realization of nanostructured sensors based on Si a strategic challenge for both scientific and industrial research. Among the various silicon nanostructures, silicon nanowires (Si NWs) are emerging as ideal materials for the design of devices in the sensors field. Due to their easy preparation and integration with a typical microelectronics’ flat architecture, simple control of their structural properties, and a very high S/V, they are stating as a powerful class of ultrasensitive sensors for the detection of different biological and chemical species.

The Si NW sensors currently present in the literature are based on the variation of their electrical properties [36,37,38] and, only recently, our group demonstrates the realization of a novel Si NW platform based on room temperature (RT) luminescence for biomarker as well as DNA analysis [39,40]. Indeed, Si is an indirect bandgap semiconductor and to obtain RT emission, it is necessary to have quantum confinement. Although light-emission has been obtained from porous silicon [41,42] and Si nanocrystals [43,44], these nanomaterials present several drawbacks that limit their sensing application such as aging effect and light-emission stability and intensity [45,46].

Several silicon nanowire sensors have already been developed for the detection of gaseous substances, and, in particular, to match the NO_2_ challenge nowadays. However, most of them involve functionalization processes or are decorated with metal nanoparticles to improve their performances in terms of electrical response [47,48,49].

Li et al. fabricated a device based on porous silicon nanowires (P-Si NWs) for the detection of NO_2_ [47]. The sensor based on bare Si NWs shows a small RT electrical response starting from 5 ppm of NO_2_ and becomes appreciable over 50 ppm, concentrations that are too high to integrate it in an environmental safety context. Moreover, it is notable that the baseline resistance does not remain constant but is always lower after each NO_2_ pulse. This suggests sensor poisoning, which makes the measure less reliable and the sensor non-recyclable. The same authors have shown better results after the deposition of zinc oxide (ZnO) nanorods (NRs) along the surface of the nanowires. Even if this does not change the LOD, the use of ZnO makes the measure more reliable with a 30% increment of the resistance response. Nonetheless, the sensor continues to show a poisoning effect with an unstable baseline resistance [50].

In the literature, a sub-ppm detection of the NO_2_ by a Si NW sensor has already been reported [51]. In this work, In et al. demonstrate the realization of a Si NWs-based device able to detect down to 10 ppb of NO_2_. These results were obtained by using a porous metal top electrode (PTE) that covers the whole nanowire’s upper surface. The same authors show the strong influence of the PTE on response times. The larger the covered area is, the longer the response times are. Despite the high performance in terms of the LOD, this sensor suffers from very high response times that, in the case of sub-ppm detection, are even longer than 30 min. Recovery times are commonly always longer than response times. The device reflects the great potential in terms of the sensitivity of Si NWs; however, the sensor is not applicable in a real framework where quick responses are demanded to reveal gases such as NO_2_ to avoid and minimize the possible health risks. This is a huge drawback, especially for the analysis of real complex matrices. Moreover, the fabrication of this type of device is complex, time-consuming, and very expensive, limiting their commercial transfer.

In contrast to the gas sensing devices based on electrical performances, optical sensors are commonly known as the most reliable gas sensing platforms due to the absence of electrical noise issues [52,53]. However, the realization of a Si NW chemical sensor based on room temperature (RT) photoluminescence (PL) is, to the best of our knowledge, completely absent in the literature. This can be ascribed to the traditional techniques used in the Si NW fabrication that make quantum confinement and PL at RT extremely complex [50,54]. In fact, the most commonly used techniques for Si NW synthesis are Vapor–Liquid–Solid (VLS) and Reactive Ion Etching coupled with lithography. However, without other more complex procedures by both of them, it is extremely complex to obtain suitable quantum confinement dimensions [55]. Indeed, as far as the authors know, no case of gas sensing devices based on Si NW light emission are reported in the literature. Recently, our group demonstrates the use of a modified Metal-Assisted Chemical Etching (MACE) process, by using ultra-thin gold (Au) films to synthesize RT light-emitting Si NWs [56] due to the quantum confinement effect, as already investigated in our works [57,58,59,60,61,62]. This approach is industrially compatible, maskless, and cost-effective. Moreover, very high densities of Si NWs (10^12^ NWs/cm^2^) can be obtained in a few minutes. By varying the thickness of the Au deposited, it is possible to control the average diameter and to obtain Si NWs with quantum confinement and PL at RT [63].

In this article, we present a novel gas sensing solution based on RT luminescent Si NWs that can be used with both electrical and optical transduction. In particular, the realization of a Si NWs-based sensor without decoration or surface functionalization will be shown for the detection of NO_2_.

## 2. Materials and Methods

### 2.1. Si NWs Sensor Synthesis

**Figure 1:**
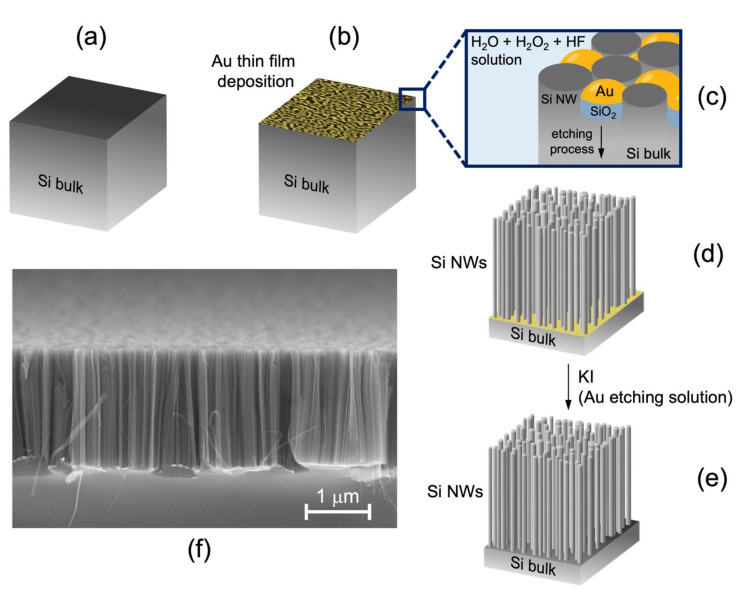
(**a**–**e**) Schematic representation of the Si NW realization process by using the MACE technique. (**f**) Scanning Electron Microscopy of Si NWs in cross section.

Si NWs were fabricated using the thin film Metal-Assisted Chemical Etching (MACE) technique in order to synthesize a high density of NWs with controlled structural and doping properties [64,65]. Si NWs were prepared starting from a (100)-oriented p++ type (~10^19^ Boron atoms/cm^3^) commercial Si wafer, as schematized in Figure 1a. At first, the silicon wafer surface was cleaned with a UV-treatment for 5 min and then by immersing the sample in a 5% hydrofluoric acid (HF) aqueous solution for 2 min in order to obtain a surface free of native SiO_2_. Subsequently, a thin film of 2 nm of gold was deposited by an electron beam evaporation at room temperature (RT), as shown in Figure 1b. Under specific deposition conditions, this Au thin layer was discontinuous and the uncovered Si areas had an average diameter of 9 ± 2 nm, as measured through the statistical analysis of different SEM images taken after the deposition of the Au layers thickness [65]. The sample was then immersed in an HF (5 M) and H_2_O_2_ (0.44 M) etching aqueous solution. Due to the greater electronegativity of gold with respect to silicon, the selective oxidation of Si by the H_2_O_2_ occurred under the Au regions. This promoted the local SiO_2_ production only in the Au covered regions. Concurrently, HF reacted with the SiO_2_, dissolving it in solution. As pictured in Figure 1c, in the Si areas covered by the metal, a selective excavation of the silicon is observed, while the formation of Si NWs follows in the uncovered Si areas. The Si NWs fabricated using this method have an average diameter of 7 ± 2 nm, as demonstrated by both Raman fitting and Energy Filtered Transmission Electron Microscopy (more details on [65]).

This process ensures a strong control on the Si NW structural characteristics. Indeed, it is possible to vary the length and the diameter of the Si NWs by changing the etching time and the thickness of the Au layer, respectively. Unlike common Vapor–Liquid–Solid techniques the whole process is performed at room temperature; therefore, gold does not diffuse inside the Si nanowires but remains on the bottom of the etch and it is removed by a KI gold etching solution for 1 min, as shown in Figure 1d,e. This method is maskless, low cost, fast, scalable on commercial wafer, and compatible with the current Si technology. Furthermore, this method allows us to realize NWs with the same doping and crystalline orientation of the starting silicon wafer.

### 2.2. Structural, Optical and Electrical Characterization

Structural characterization of Si NWs was carried out by a Scanning Electron Microscopy (SEM) using a field emission Zeiss Sigma microscope (Carl-Zeiss-Straße 22, 73447 Oberkochen, Germany) (5 kV, 30 μm of aperture). Figure 1f reports a cross section SEM image of the synthesized Si NWs, showing a very dense vertically aligned array of ~10^12^ NWs/cm^2^.

As already discussed in Section 1, these Si NWs emit light at room temperature by quantum confinement effect. Photoluminescence (PL) spectra were collected at room temperature using a HR800 spectrometer (Horiba Jobin Yvon, HORIBA, Ltd. Head Office/Factory 2, Miyanohigashi, Kisshoin Minami-Ku Kyoto 601-8510, Japan) coupled to a cooled CCD detector focusing 100 μW of the 476 nm line of an Ar^+^ laser onto the sample plane. Each PL spectrum shown is the result of a statistical average of numerous measurements (at least six) recorded on the whole surface of the sample. PL spectra of the sensor in the presence of gas were acquired by using a setup that generates and transports controlled flows of gas to the sensor. The acquisition time is of a few seconds. The sample is placed in a closed cell where the sensors’ performance at a controlled temperature and in the presence of known concentrations of the target gas are tested.

The used NO_2_ gas target is contained within permeation tubes where the quantity of gas that permeates from the membrane of the tube depends on the permeation rate through the tube membrane and on the temperature to which it is subjected. Both optical and electrical measurements were conducted by heating the permeation tube to 50 °C.

Electrical measurements were carried out by using the same instrument and the electrical response of the sensor was measured with a digital multimeter (set to generate a constant voltage of 2 V) connected to a raspberry pi. The electrical contact between the electrodes of the digital multimeter and the sensor was achieved by depositing 100 nm of Au onto 5 nm of titanium (Ti) through the use of specific aluminum (Al) masks. Au deposition was carried out immediately after Ti deposition without breaking the vacuum to avoid Ti oxidation. The chamber was maintained in high vacuum condition of about 10^−6^ mbar for all the depositions. The electrical contact realization on top of the Si NWs is also compatible with the current industrial silicon methodologies.

### 2.3. Materials

Commercial Si wafers were purchased from Siegert Wafer (SIEGERT WAFER GmbH Charlottenburger Allee 7 · 52068 Aachen, Germany). Reagents used for the synthesis of Si NWs as HF (48%), H_2_O_2_, and KI (gold etchant standard) were purchased from Sigma Aldrich (Merck KGaA Headquarters of the Merck Group Frankfurter Strasse 250 Darmstadt, 64293, Germany). NO_2_ permeation tubes were purchased from Fine Metrology srls (Via Vincenzo Monti 14, 98048, Spadafora, Messina, Italy).

## 3. Results and Discussion

Si NWs prepared using the previously discussed MACE technique have been tested as a novel gas sensing platform. In particular, we have focused our efforts on the detection of nitrogen dioxide, which is a forefront field for the development of increasingly sensitive and efficient sensors in monitoring the safety of both urban and closed environments. We will show the electrical and optical performance in the presence of NO_2_.

### 3.1. Optical Measurements

**Figure 2:**
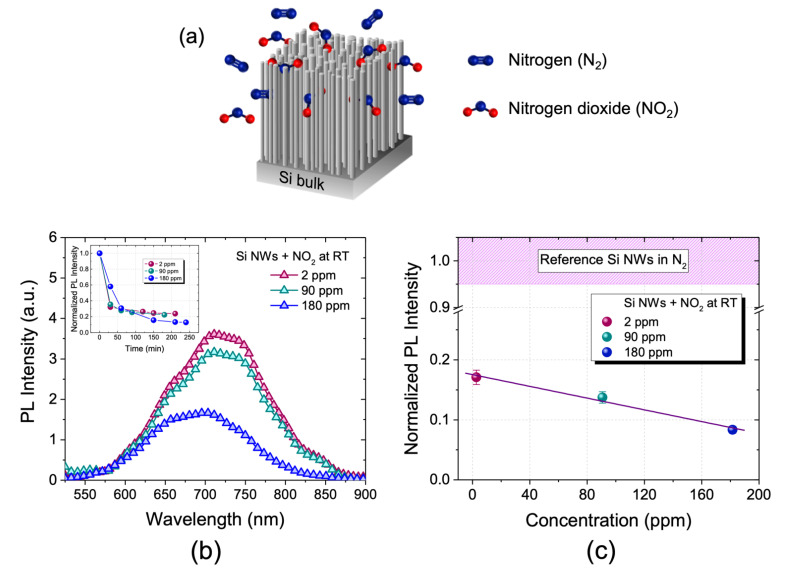
(**a**) Schematic representation of the Si NW sensor in the presence of the N_2_/NO_2_ gas mixture. (**b**) RT photoluminescence spectra of the sensor exposed to 2 ppm NO_2_ (red line), 90 ppm NO_2_ (green line), and 180 ppm NO_2_ (blue line). (**c**) Calibration curve of the sensor at room temperature.

In optical measurements, the sample is initially exposed to a controlled flow of nitrogen (N_2_) in order to provide a free interfering substances chamber. After that, a mixture of N_2_ and NO_2_ is blown into the chamber for a specified time (until there is no further PL variation for optical measurements) and at different tested working temperatures. The gases infiltrate within the dense 3D array and interact with the Si NWs, as schematized in Figure 2a, causing the change in optical properties.

Since the realization of light-emitting Si NWs at room temperature was just recently achieved, the use of their PL as a gas sensing transduction has never been applied to the best of our knowledge. First of all, we tested the Si NW PL response in the presence of a controlled amount of NO_2._ In Figure 2b, the RT PL spectra obtained by testing the sensor to a concentration of 2, 90, and 180 ppm of NO_2_ are reported. These spectra show the typical Si NW emission band between 550 and 900 nm due to quantum confinement.

In particular, the sensor was exposed to a controlled flow of 350 cc/min of N_2_ for one night in order to obtain an inert environment. Subsequently, it was exposed to a controlled flow of the N_2_/NO_2_ mixture corresponding to the following different concentrations of NO_2_: 2 ppm (red line), 90 ppm (green line), and 180 ppm (blue line). As can be seen from Figure 2b, by increasing the concentration of NO_2_ the photoluminescence of the sensor decreases. The gas interaction probably causes the introduction of new non-radiative levels; therefore, the system is deactivated in its ground state, dissipating energy without photon emission. Each spectrum was acquired at room temperature by exposing the sensor to that concentration of the gas mixture. In particular, these RT PL spectra were acquired every 30 min until no signal variation was observed. The inset to Figure 2b shows the Si NW sensor’s normalized PL as a function of the measurement time for all of the three NO_2_ concentrations (2, 90, and 180 ppm). All the curves show the same saturation trend characterized by a first very fast linear phase in which the signal reaches more than 90% of the final value (within the first hour) and a second slower, asymptotic phase, in which the sensor’s stabilization is observed. As can be seen, by increasing the NO_2_ concentration, the sensor’s stabilization is achieved at lower PL values.

In order to accurately estimate the variation of photoluminescence intensity of Si NWs for the different NO_2_ concentrations, each spectrum was fitted using a single Gaussian model. The red spectrum represents the PL of the sensor exposed to 2 ppm of NO_2_, this signal decreases by a factor of 1.2 when the concentration is increased to 90 ppm (green spectrum) and still decreases by a factor of 1.6 when the concentration is increased to 180 ppm (blue spectrum). The latter spectrum has a different line shape than the previous ones and also the maximum emission is blue-shifted. This trend is probably due to the introduction of novel non-radiative levels by the physisorption of NO_2_ that causes the quench of the PL. This mechanism has already been demonstrated for optical biosensors based on these Si NWs, where a higher concentration of the target corresponds to a higher PL quenching [39,40].

In Figure 2c, the calibration curve of the sensor is reported. The signal reference is related to the fitted photoluminescence intensity of the Si NWs exposed only to N_2_ flow. The red dot represents the PL of the sensor exposed to 2 ppm of NO_2_ that decreases by about 83% compared to the reference signal. The green dot is related to the PL of the sample exposed to 90 ppm of NO_2_ with a variation of about 86% compared to the reference signal. Finally, the blue dot represents the PL of the sample exposed to 180 ppm of gas with a variation of about 92% compared to the reference signal. This region of concentration is characterized by a linear decreasing PL trend, as demonstrated by the linear fit in Figure 2c. The straight line passes through the experimental points (with an error of ± 7% each) and the fit is associated with an r^2^ of 0.98 and a Pearson coefficient of 0.99, which guarantee a reliable fit in the analyzed region.

It is clearly shown how the sensor is sensitive not only to gas variations but also to very small quantities of NO_2_, on the order of a few ppm. This is due to the very high surface to volume ratio of these Si NWs and, therefore, to a strong interaction with the substances with which they interact. We were able to obtain a low limit of detection with a cost-effective Si-based system, without any functionalization, at room temperature. The sensor’s LOD is expected to be improved through surface functionalization with molecules capable of selectively binding NO_2_ and this should also ensure greater sensitivity in shorter times.

### 3.2. Electrical Measurements

**Figure 3:**
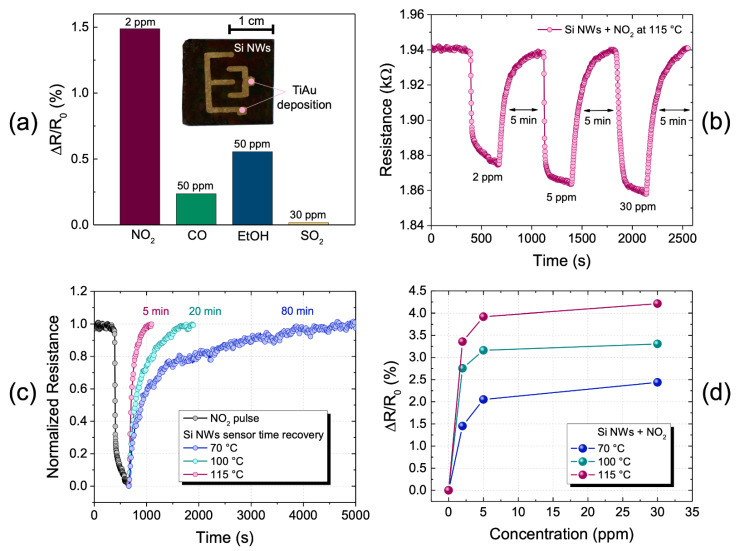
(**a**) photograph of the sensor (2 × 2.3 cm^2^) made up of Si NWs (black color) covered by the interdigitated electrode of 100 nm Au above 5 nm of Ti. (**b**) Measurement of electrical resistance of the sensor exposed to 2, 5, and 30 ppm of NO_2_ pulses at 115 °C. (**c**) Sensor recovery times after 5 min of NO_2_ pulse at 2 ppm at 70, 100, and 115 °C. (**d**) Variation of the sensor resistance as a function of the NO_2_ concentration at 70, 100, and 115 °C.

The picture inside Figure 3a shows a photograph of the Si NW sensor on which 100 nm of Au metal layer was deposited over 5 nm of Ti adhesion layer with an interdigitated geometry. The system of two interdigitated electrodes placed above the Si NWs has a macro dimension compared to the standard microelectronics systems where electrodes are realized through photolithography mask processes.

In nanostructured materials, the electrical conduction is strongly affected by surface phenomena [66]. The use of an interdigitated electrode geometry on top of the Si NWs allows the conduction across the whole surface of nanowires to be exploited and their resistance without the substrate influence to be measured.

The graph in Figure 3a shows the comparison of the sensor electrical responses with different gases at the working temperature of 70 °C. In particular, the variation of sensor resistance, following 2 ppm of NO_2_, 50 ppm of CO, 50 ppm of EtOH, and 30 ppm of SO_2_ is reported. The resistance variation shown here, as those below, was calculated by using Equation (1), as follows:

(1) $$\frac{\Delta R}{R_{0}}\left(\%\right)=100\frac{R_{0}-R}{R_{0}}$$

where *R*_0_ is the sensor resistance (baseline), and *R* is sensor resistance exposed to NO_2_ (pulse resistance).

The resistance variations were calculated following 5-minute-long gas pulses for all the measurements. As can be seen from the histogram, the sensor’s response to nitrogen dioxide at 2 ppm (our minimum tested concentration) is significantly higher (more than three times) than the responses to the other gases, despite their concentrations being much higher. This is a clear confirmation of the Si NWs-based sensor selectivity towards NO_2_ compared to the other tested gases. To verify the sensor response to nitrogen dioxide, gas sensing measurements were made both in the presence of different concentrations of NO_2_ and at different temperatures. Three scans were carried out at three different temperatures (70, 100, and 115 °C) in which the resistance of the sensor was measured at three NO_2_ concentrations (2, 5, and 30 ppm).

In particular, the sensor was exposed to a controlled flow of 350 cc/min of N_2_ for one night to obtain an inert environment. Subsequently, it was exposed to a controlled flow of the N_2_/NO_2_ mixture corresponding to the following different concentrations of NO_2_: 100 cc/min, which corresponds to about 2 ppm of NO_2_; 50 cc/min, which corresponds to about 5 ppm of NO_2_; and 10 cc/min, which corresponds to about 30 ppm of NO_2_. For all three concentrations, a NO_2_ permeation tube with a permeation rate of 507 ng/min was used.

Figure 3b shows the complete profile of the electrical response of the sensor at 115 °C. The first part of the curve is related to the stabilization of the sensor under an overnight constant N_2_ flow (350 cc/min). The sensor was stable in these operating conditions with a resistance of 1.940 kΩ. After that, NO_2_ pulses at different concentrations were introduced into the chamber for 5 min each. All the gas pulses are characterized by an initial descending trend with a very high slope relative to the interaction of the sensor with the gas, a second regime in which the slope of the curve decreases over time, indicating that the gas is saturating the sensor surface, and finally a third part, where the sensor degasses returning to its baseline in the presence of only N_2_. By using 2 ppm of NO_2_, the resistance drops to 1.874 kΩ. The resistance change compared to its baseline (∆*R/R*_0_) is about 3.4% with a recovery time of 5 min.

In the presence of 5 ppm NO_2_, the resistance drops to 1.864 kΩ with a variation of about 3.9% and a recovery time of 5 min. In the presence of 30 ppm of NO_2_, the resistance drops to 1.858 kΩ with a variation of about 4.2% and a recovery time of 5 min. As expected, we found the Si NW sensor recovery time-dependent on the temperature of the sensor, since it refers to the physical-chemical interaction of the gas on the Si NW surface. In fact, as shown in Figure 3c, the recovery time varies according to the working temperature. In order to show this phenomenon, in this figure the first pulse of 2 ppm of NO_2_, normalized for all the studied temperatures of 70, 100, and 115 °C, is reported. It is clearly visible that the recovery time decreases drastically by increasing the temperature. At 70 °C, the sensor shows a recovery time of 80 min. When the temperature is increased to 100 °C, the time is lowered to 20 min, and by increasing the temperature to 115 °C, it is reduced to 5 min. The well-aligned nanowires lead to open and regular channels, which act as effective transport paths for gas molecules diffusion. Therefore, favoring the desorption of NO_2_ molecules from the active sites by a slight increase in the temperature, these can be effectively adsorbed, and then easily swept away from the sensitive layer. This is a very interesting aspect because by lightly increasing the working temperatures, the recovery times are greatly reduced and, therefore, it is possible to obtain faster and easier sensor recyclability.

Figure 3d shows the percentage resistance changes (∆*R/R*_0_) as a function of each NO_2_ pulse concentration at each working temperature of 70, 100, and 115 °C. All the curves show the same trend. The resistance variation increases by increasing both the NO_2_ concentration and the temperature. It is well known that NO_2_ is a strong oxidizing gas, which acts as electrons’ acceptor. Whenever the Si NW sensor is exposed to NO_2_, this can be directly adsorbed onto the sensing surface trapping electrons, or interact with the chemisorbed oxygen species, further subtracting additional electrons from the bulk [67]. In particular, it was found that NO_2_ adsorption consists in forming different surface nitrogen-containing molecular groups and dangling bonds of Si atoms on the surface [68]. This interaction, in turn, influences the electrical conductivity and, consequently, the sensing properties towards this reactive species. However, due to the stabilization of the sensor in an inert environment, the presence of an adsorbed oxygen species can be excluded, then the direct interaction of NO_2_ molecules with Si NWs can be assumed as the main effect of the sensing mechanism. The silicon substrate used for the fabrication of the sensor is a p-type material, in which holes (*h*^+^) are the major charge carriers involved in its electrical conduction mechanism. Therefore, the adsorption of NO_2_ molecules, capturing free electrons at the surface, leads to the release of trapped holes into the conduction band of NWs, then to the decrease in sensor resistance. The related kinetic reaction can be represented as Equation (2), as follows:

(2) $${\mathrm{NO}}_{2}\left(\mathrm{gas}\right)+e^{-}\to {\mathrm{NO}}_{2}^{-}\;\left(\mathrm{ads}\right)+h^{+}$$

The higher the number of adsorbed NO_2_ molecules by an increased gas concentration in the environment is, the larger the concentration of free holes in the conduction band, and then the resistance variation of the sensor, is. The sensor seems to saturate at high concentrations (>5 ppm), and this is evident from the change in the slope that the curve assumes (as shown by the 30-ppm measure) for all the temperatures. This trend suggests that by increasing the temperature, the sensor is able to measure lower concentrations, maintaining a high signal-to-noise ratio (>42 in the best case of 115 °C). This very important aspect is a key element for the detection of life-hazard concentrations around 2–3 ppm.

Table 1 reports the electrical performances of our Si NW sensor compared to the literature of the NO_2_ sensing.

As it is possible to observe the good performances in terms of the LOD and recovery time, here reported by the Si NW sensor, even if detrimentally affected by the macro contact, which are already competitive with the literature. More importantly, these measures show a tremendous increase potentiality for electrical contact scaling, paving the way to a novel Si-based and complementary metal-oxide-semiconductor (CMOS) compatible cost-effective gas microsensor.

## 4. Conclusions

We demonstrated the realization of a gas sensing device based on Si NWs. The peculiar properties of these Si NWs have been investigated for the optical and electrical recognition of NO_2_, an extremely dangerous gas already at low concentrations. The sensor shows remarkable responses to different NO_2_ concentrations up to 2 ppm through both PL and resistance variations. The possibility of using the same sensor for both photoluminescence and electrical analysis makes the platform highly versatile and, therefore, usable for multiple categories of operators.

The evident changes in PL and resistance already at 2 ppm of NO_2_ suggest that the sensor can respond to even lower concentrations. By considering the size of the sample used, it is reasonable to observe recovery times that can be shortened, especially for the electrical measurements where the interdigitated has millimeter features. However, it has been shown that small temperature variations shorten the recovery time much more. Therefore, by varying the temperature and with the miniaturization of the sensor, it may be possible to decrease the recovery times to improve the sensor’s performances. These measurements are an interesting starting point for the realization of novel Si NW sensors that couple very high performances with an industrially compatible and low-cost process. All of these points are widely demanded for the realization of large-scale diffused commercial devices that may have a serious impact on human life and environmental control.

## Figures and Tables

**Table 1:** Comparison of NO_2_ sensing properties of our Si NWs-based chemoresistive sensor with other sensors.

Sample	NO_2_ (ppm)	Temperature (°C)	Response time (s)	Recovery Time (s)	Ref.
Si NWs	2	115	75	300	This work
Cr_2_O_3_	3	200	-	-	[69]
Fe_2_O_3_	1	225	26	48	[70]
Cu:Fe_2_O_3_	5	300	85	105	[71]
α-Fe_2_O_3_	1	200	77	120	[72]
ZnO NRs/P-Si NWs	5	RT	800	780	[47]
Si NWs	0.01	40	-	-	[51]
ZnO NWs	2	200	1200	1200	[73]
In_2_O_3_	0.5	100–250	>170	>120	[74]
SnO	0.5	200	90	80	[75]
TeO_2_	10	RT	660	1200	[76]

## Data Availability

Data are contained within the article.
